# CNPY2 Aggravates Renal Tubular Cell Ferroptosis in Diabetic Nephropathy by Regulating PERK/ATF4/CHAC1 Pathway and MAM Integrity

**DOI:** 10.1002/advs.202416441

**Published:** 2025-04-11

**Authors:** Jingfang Chen, Dongwei Liu, Lei Lei, Ting Liu, Shaokang Pan, Hui Wang, Yong Liu, Yingjin Qiao, Zhangsuo Liu, Qi Feng

**Affiliations:** ^1^ Department of Nephrology, the First Affiliated Hospital of Zhengzhou University Research Institute of Nephrology, Zhengzhou University Traditional Chinese Medicine Integrated Department of Nephrology，the First Affiliated Hospital of Zhengzhou University Henan Province Research Center for Kidney Disease，the First Affiliated Hospital of Zhengzhou University Key Laboratory of Precision Diagnosis and Treatment for Chronic Kidney Disease in Henan Province the First Affiliated Hospital of Zhengzhou University Zhengzhou 450052 P. R. China; ^2^ Department of Cardiology Henan Provincial Chest Hospital Chest Hospital of Zhengzhou University Zhengzhou 450003 P. R. China; ^3^ Tianjian Laboratory of Advanced Biomedical Sciences Academy of Medical Sciences Zhengzhou University Zhengzhou 450001 P. R. China; ^4^ Innovation Center of Basic Research for Metabolic‐Associated Fatty Liver Disease Ministry of Education of China, P. R. China; ^5^ Department of Cardiology the First Affiliated Hospital of Zhengzhou University Zhengzhou 450052 P. R. China

**Keywords:** canopy FGF signaling regulator 2 (CNPY2), diabetic nephropathy, endoplasmic reticulum (ER) stress, ferroptosis, mitochondria‐associated endoplasmic reticulum membrane (MAM)

## Abstract

Ferroptosis is emerging as a novel mechanism for understanding renal tubular injury in diabetic nephropathy (DN). The mitochondria‐associated endoplasmic reticulum membrane (MAM) plays a crucial role in the regulation of numerous cellular processes, including mitochondrial dysfunction and endoplasmic reticulum (ER) stress (ERS). However, the exact mechanism underlying ferroptosis and MAM in DN remains unclear. In this study, we identified that canopy FGF signaling regulator 2 (CNPY2) is upregulated in the renal tubules of DN. Downregulation of CNPY2 alleviated ferroptosis and improved MAM integrity in the renal tubular epithelial cells of db/db mice. Conversely, CNPY2 overexpression aggravated tubular injury in DN by accelerating ferroptosis and disrupting MAM formation. Mechanistically, CNPY2 activated the PERK/ATF4/CHAC1 signaling pathway to facilitate ferroptosis, thus contributing to tubular injury in DN. These findings highlight the critical role of CNPY2 in modulating ferroptosis and MAM formation in DN progression, and suggest that CNPY2 is a feasible therapeutic target for DN.

## Graphical abstract

Canopy FGF signaling regulator 2 (CNPY2) which is contributed to ERS is upregulated in the renal tubules of patients with diabetic nephropathy (DN). Mechanistically, CNPY2 may regulate ferroptosis and MAM integrity in renal tubular epithelial cells through PERK/ATF4/CHAC1 pathway, thereby promoting the progression of DN.

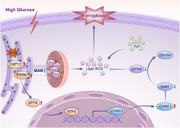

## 1 Introduction

Diabetic nephropathy (DN) is a prevalent and severe microvascular complication of diabetes and the leading cause of end‐stage renal disease.^[^ ^1^, ^2^ ^]^ Approximately 30–40% of people with diabetes eventually develop DN worldwide.^[^ ^3^ ^]^ The pathogenesis of DN is complex and has not been fully elucidated, but the key role of tubular injury has attracted increasing attention.^[^ ^4^, ^5^ ^]^ Prior researches have indicated a strong association between tubulointerstitial fibrosis and the progression of DN, suggesting that damage to renal tubular epithelial cells (RTECs) occurs at an early stage of DN, even preceding the onset of microalbuminuria.^[^ ^6^, ^7^ ^]^ However, the exact mechanism underlying the tubular injury in DN remains unclear.

Ferroptosis is a distinct form of programmed cell death characterized by iron‐dependent lipid peroxidation and accumulation of reactive oxygen species (ROS).^[^ ^8^, ^9^ ^]^ Previous studies have established the pivotal role of ferroptosis in renal tubular damage in individuals with diabetes.^[^ ^10^, ^11^, ^12^ ^]^ Our previous investigation^[^ ^13^ ^]^ revealed characteristic changes in ferroptosis, such as ferrous iron deposition, glutathione (GSH) depletion, elevated lipid peroxidation levels, and mitochondrial ridge reduction in the renal tubules of db/db mice. Consequently, elucidating the molecular pathways underlying ferroptosis in RTECs is critical for understanding the progression of DN.

Aberrant activation of endoplasmic reticulum (ER) stress (ERS) sensors and their downstream pathways has emerged as a critical regulatory mechanism in DN.^[^ ^14^ ^]^ Liu et al. demonstrated that ERS is activated in RTECs under high‐glucose conditions, leading to ferroptosis via the XBP1‐Hrd1‐Nrf2 signaling pathway.^[^ ^15^ ^]^ The mitochondria‐associated endoplasmic reticulum membrane (MAM) serves as a dynamic interface between the outer mitochondrial membrane and the ER, playing a vital role in maintaining mitochondrial dynamics, ERS, Ca^2+^ homeostasis, glucose metabolism, and autophagy.^[^ ^16^ ^]^ In a high‐glucose environment, overexpression of Reticulon‐1A (RTN1A) in RTECs activates ERS and impairs mitochondrial function by modulating MAM.^[^ ^14^ ^]^

Canopy fibroblast growth factor (FGF) signaling regulator 2 (CNPY2) is localized in the ER and serves as a significant regulatory factor in ERS.^[^ ^17^ ^]^ Previous studies have indicated that CNPY2 plays a crucial role in activating the protein kinase R (PKR)‐like ER kinase (PERK) pathway in response to unfolded proteins in the ERS, thereby influencing cellular processes such as cell growth, migration, and apoptosis.^[^ ^17^ ^]^ However, the specific role of CNPY2 in the pathogenesis of DN remains unclear.

In this study, we demonstrated that the expression of CNPY2 was increased in the renal tubules of patients with DN and db/db mice. Furthermore, the knockdown of CNPY2 alleviated ferroptosis and improved MAM formation in the renal tubules of db/db mice. Conversely, CNPY2 overexpression worsened tubular injury in DN by accelerating ferroptosis and disrupting MAM integrity. Moreover, in a high‐glucose environment, CNPY2 partially regulated the PERK/ATF4/CHAC1 signaling pathway to facilitate ferroptosis in RTECs, thereby contributing to tubular damage in DN. To our knowledge, this is the first study to report the potential role and mechanism of CNPY2‐mediated tubular injury in DN.

## 2 Results

### 2.1 CNPY2 is Notably Upregulated in RTECs from Patients with DN and db/db Mice

**Figure 1:**
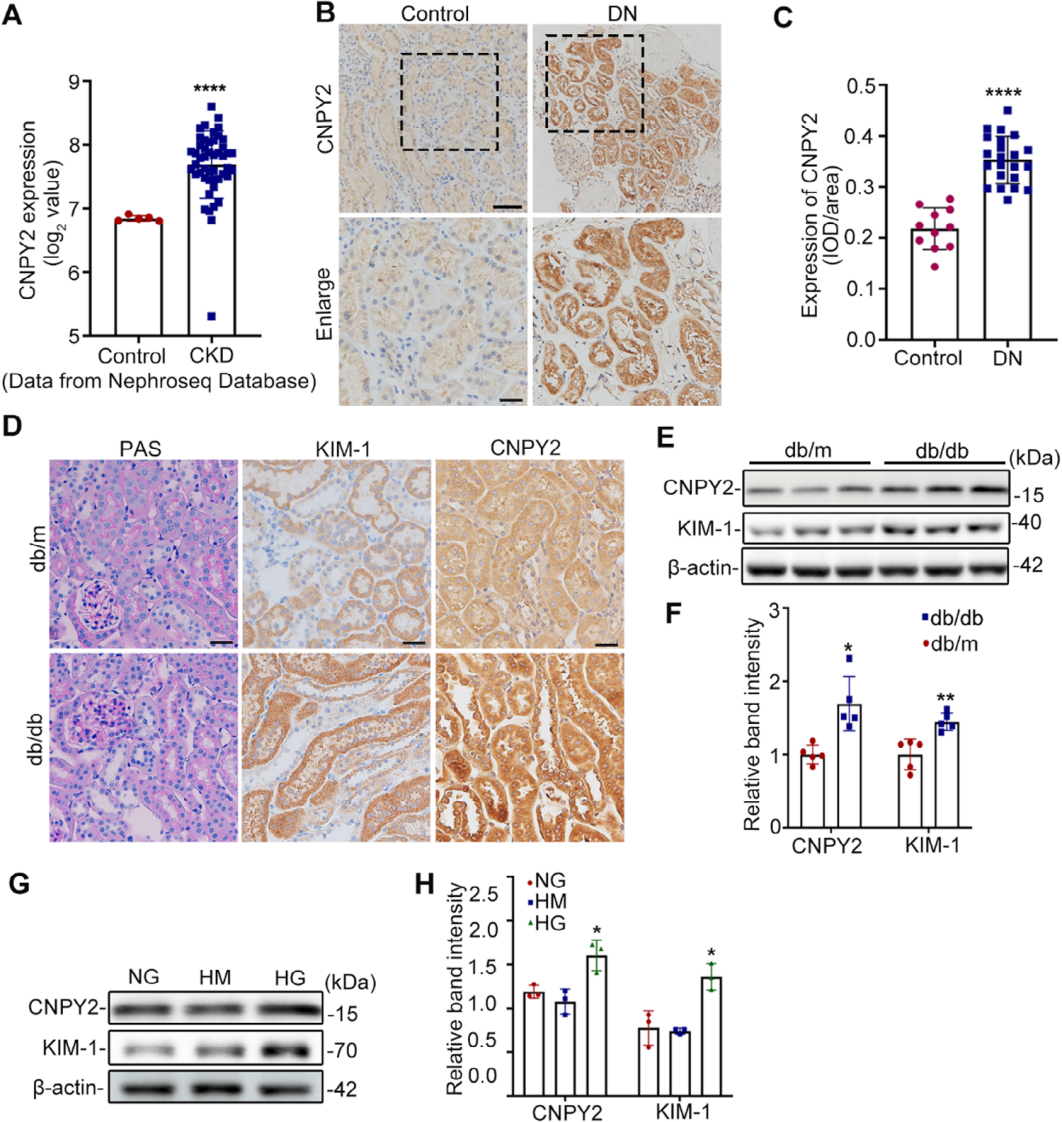
CNPY2 is notably upregulated in RTECs from patients with DN and db/db mice. A) Analysis of CNPY2 expression in the kidney tissues of patients with CKD using data from the Nephroseq Database. B) Representative images of IHC staining in the kidney tissues from patients with DN (n = 22) and controls (n = 11). Scale bars, 100 µm (upper panel) and 50 µm (lower panel). C) Quantification of CNPY2 expression in IHC samples. D) PAS and IHC staining of kidney tissues from db/m and db/db mice. E) Representative western blot bands of CNPY2 and KIM‐1 in the kidney cortices of db/m and db/db mice. F) Quantitative analysis of CNPY2 and KIM‐1 levels in (E), n = 5. G,H) Western blot bands (G) and quantitative analysis (H) of CNPY2 and KIM‐1 in HK‐2 cells stimulated with 5.6 mM glucose (NG), mannitol (HM) or 30 mm glucose (HG), n = 3. All the data are expressed as the mean ± SD. ^*^ *P* < 0.05, ^**^ *P* < 0.01, ^****^ *P* < 0.0001. *P* value was calculated using two‐tailed unpaired *t*‐test (C, F) or one‐way ANOVA followed by Tukey's test (H).

We investigated the expression of CNPY2 in kidney tissues from patients with chronic kidney disease (CKD) and healthy controls using the Nephroseq database (https://www.nephroseq.org). Analysis of the Nakagawa CKD dataset revealed significant upregulation of CNPY2 in the kidney tissues of patients with CKD compared to normal controls (**Figure** **1**A). To further assess the potential role of CNPY2 in DN, we examined the expression levels of CNPY2 in renal biopsy tissue samples from patients with DN and control participants using immunohistochemical (IHC) staining. The results indicated that CNPY2 expression was significantly upregulated in the RTECs of the DN group compared to the control group (Figure 1B,C) as well as in the RTECs of db/db mice (Figure 1D; Figure S1C‐F Supporting Information). Moreover, co‐immunostaining showed that CNPY2 was mainly located in the RTECs (Figure S1A, Supporting Information). And the cellular immunofluorescence colocalization assay confirmed that CNPY2 colocalized with the ER marker (calnexin, green) in HK‐2 cells (Figure S1B, Supporting Information). Western blot analysis further confirmed the upregulation of CNPY2 in the renal tissues of db/db mice, which was concomitant with increased levels of kidney injury molecule‐1 (KIM‐1), a typical marker of kidney tubular injury (Figure 1E,F). Consistently, CNPY2 expression was elevated in HK‐2 cells exposed to high glucose conditions (30 mM), accompanied by an increase in KIM‐1 expression (Figure 1G,H). Collectively, these findings indicated that CNPY2 was increased in RTECs of DN, suggesting the potential involvement of CNPY2 in the pathogenesis of DN.

### 2.2 The Level of Ferroptosis is Increased in the RTECs from Patients with DN and db/db Mice

**Figure 2:**
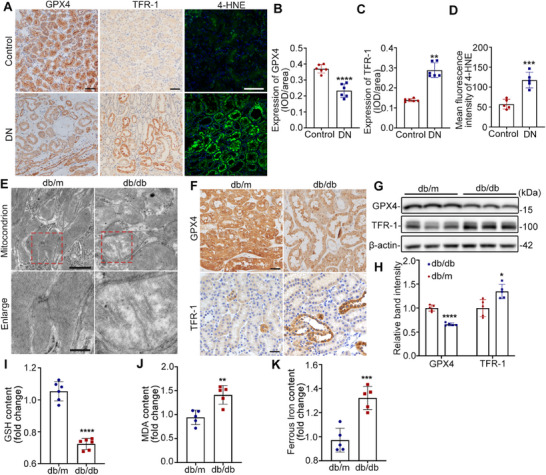
The level of ferroptosis is increased in RTECs from patients with DN and db/db mice. A) Representative expression of GPX4, TFR‐1, and 4‐HNE in the kidney tissues of patients with DN (n = 22) and controls (n = 11). Scale bars, 100 µm (upper panel) and 50 µm (lower panel). B–D) Quantification of GPX4, TFR‐1, and 4‐HNE expression in A. E) Representative TEM images showing the mitochondrial morphology in the kidneys of db/m and db/db mice. Scale bars, 1 µm (upper panel) and 500 nm (lower panel). F) Representative expression of GPX4 and TFR‐1 in the kidney tissues of db/m and db/db mice. Scale bar = 50 µm. G,H) Western blot bands (G) and quantitative analysis (H) of GPX4 and TFR‐1 in the kidney tissues of db/m and db/db mice. I–K) The contents of GSH, MDA, and ferrous iron in the kidneys of db/m and db/db mice (n = 5‐6). All the data are expressed as the mean ± SD. ^*^ *P* < 0.05, ^**^ *P* < 0.01, ^***^ *P* < 0.001, ^****^ *P* < 0.0001. *P* value was calculated via two‐tailed unpaired *t*‐test.

Recently, the potential role of ferroptosis in renal tubule injury during DN has attracted significant attention.^[^ ^18^ ^]^ Our study further verified the level of ferroptosis in the RTECs of patients with DN and db/db mice. As depicted in **Figure** **2**A–D, the expression of glutathione peroxidase 4 (GPX4) was decreased, and the expression of transferrin receptor protein 1(TFR‐1) and 4‐Hydroxynonenal (4‐HNE was significantly increased in patients with DN compared to control subjects. Transmission electron microscope (TEM) revealed alterations in the mitochondrial morphology during ferroptosis. In db/db mice, the cristae of the mitochondria almost disappeared (Figure 2E). Similarly, significant downregulation of GPX4 and upregulation of TFR‐1 were observed in db/db mice using IHC and western blot (Figure 2F–H). Moreover, GSH content decreased, and MDA and ferrous iron contents increased in the kidney tissues of db/db mice compared to those of db/m mice (Figure 2I–K).

### 2.3 MAM Formation is Disrupted in RTECs from Patients with DN and db/db Mice

**Figure 3:**
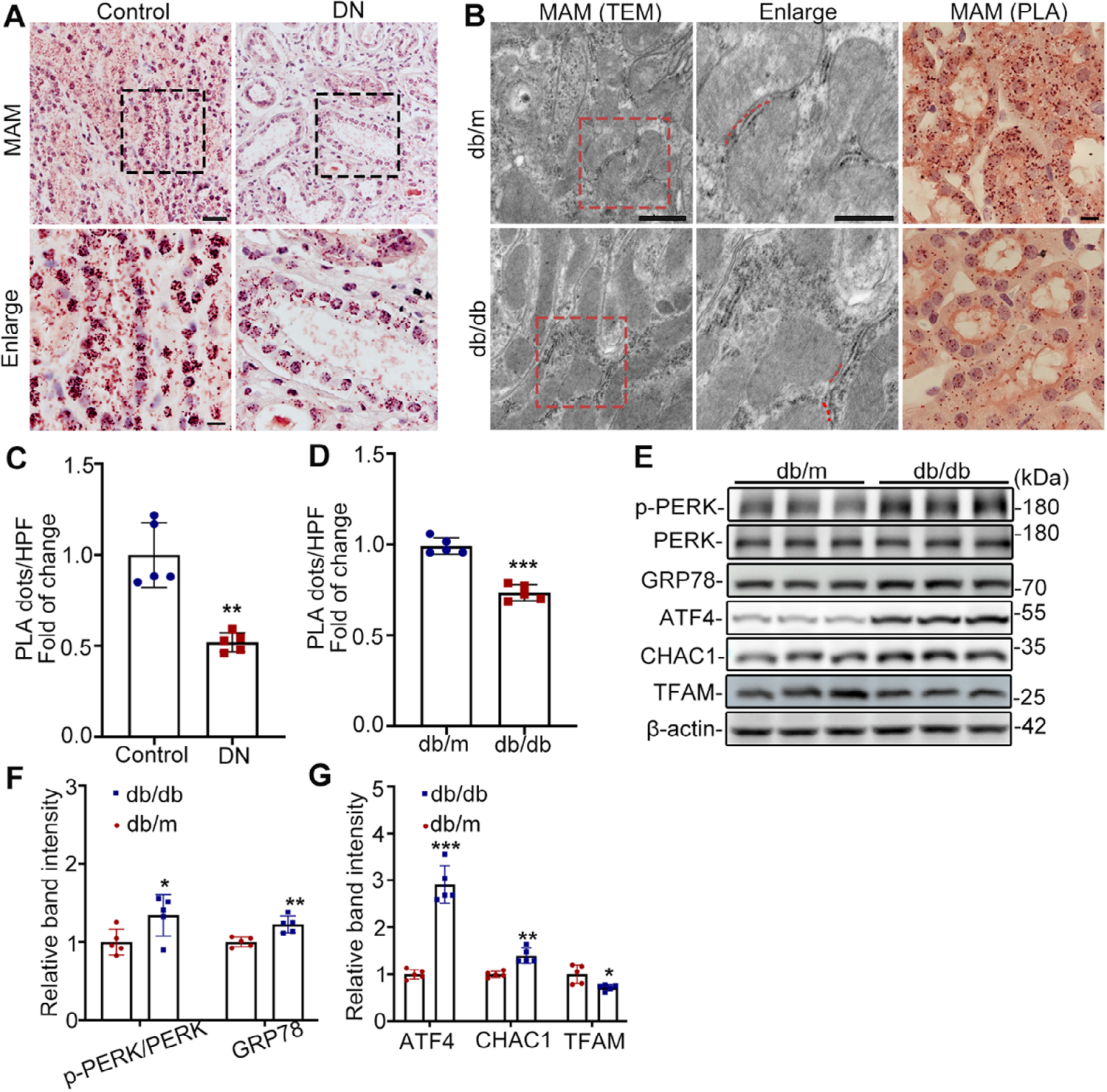
MAM formation is disrupted in RTECs from patients with DN and db/db mice. A) Representative images of MAM (PLA) in the kidney tissues of patients with DN and controls (n = 5). Scale bars, 50 µm (upper panel) and 10 µm (lower panel). B) TEM images showing the MAM area (scale bars, 50 µm (left panel) and 10 µm (right panel)) and the PLA images representing the number of MAM (Scale bar = 10 µm). C,D) Quantification of MAM in the PLA images shown in (A) and (B), n = 5. E‐G) Western blot bands (E) and quantitative analysis F,G) of p‐PERK, PERK, GRP78, ATF4, CHAC1 and TFAM in the kidneys of db/m and db/db mice, n = 5. All the data are expressed as the mean ± SD. ^*^ *P* < 0.05, ^**^ *P* < 0.01, ^***^ *P* < 0.001. *P* value was calculated using two‐tailed unpaired *t*‐test.

To gain insight into the role of MAM in RTECs from patients with DN and db/db mice, Proximity Ligation Assay (PLA) was performed to determine the number of MAM. As shown in **Figure** **3**A,C, the number of MAM decreased significantly in the RTECs of patients with DN. Similar results were obtained in db/db mice (Figure 3B,D). Consistent with the PLA results, TEM revealed a reduction in direct contact between the mitochondria and ER in db/db mice. Based on the involvement of CNPY2 in ERS, we detected ERS‐related proteins by western blot. The results revealed significantly increased expression of ERS‐related proteins, including p‐PERK, glucose‐regulated protein (GRP78), activating transcription factor 4 (ATF4), and chac glutathione specific gamma‐glutamylcyclotransferase 1 (CHAC1), in the kidney tissues of db/db mice compared with those of db/m mice (Figure 3E–G); otherwise, the mitochondrial marker protein TFAM (mitochondrial transcription factor A) showed the opposite trend in db/db mice. These findings concerning MAM alterations in DN are consistent with those of previous studies.^[^ ^19^ ^]^

### 2.4 Overexpression of CNPY2 Aggravates Ferroptosis and MAM Destruction in RTECs of db/db Mice

**Figure 4:**
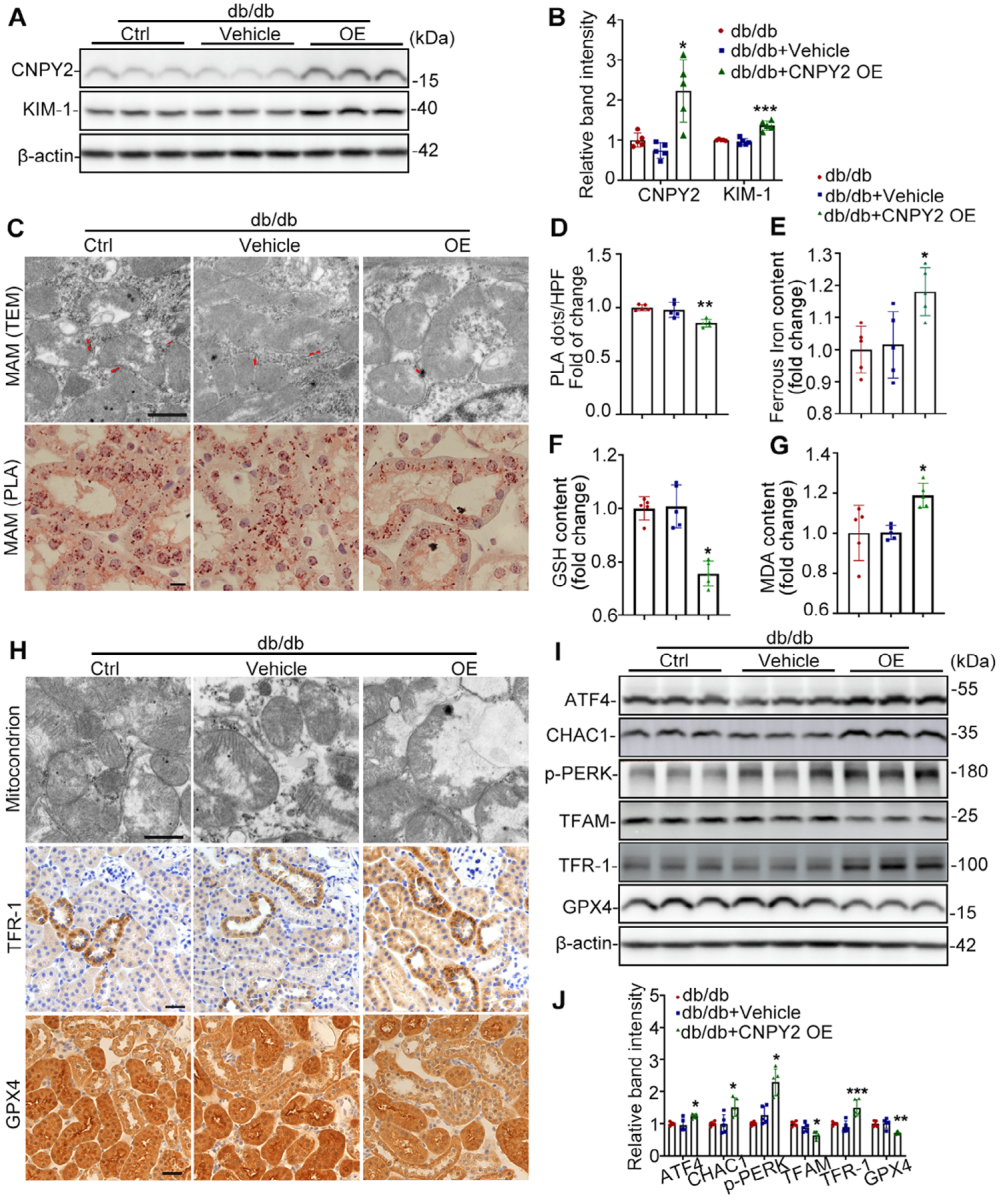
Overexpression of CNPY2 aggravates ferroptosis and MAM destruction in RTECs of db/db mice. A) Western blot bands of CNPY2 and KIM‐1 in the kidneys of db/db mice. B) Quantitative analysis of CNPY2 and KIM‐1 levels in (B), n = 5. C) Representative images of TEM and PLA showing the MAM area. Scale bars, 1 µm (upper panel) and 10 µm (lower panel). D) Quantification of MAM in the PLA images shown in (C), n = 5. E–G) The contents of ferrous iron, GSH and MDA in the kidneys of db/db mice (n = 5). H) TEM images showing the mitochondrial morphology and IHC staining showing the expression of TFR‐1 and GPX4 in the kidneys of db/db mice. I,J) Western blot bands(I) and quantitative analysis (J) of ATF4, CHAC1, p‐PERK, TFAM, TFR‐1, and GPX4 in the kidneys of db/db mice, n = 5. All the data are expressed as the mean ± SD. ^*^ *P* < 0.05, ^**^ *P* < 0.01, ^***^ *P* < 0.001. *P* value was calculated using one‐way ANOVA followed by Tukey's test.

To further explore the mechanism by which CNPY2 regulates renal tubular injury in DN, db/db mice were injected with CNPY2‐overexpressing AAV (db/db+CNPY2 OE) through the tail vein.Immunofluorescence staining showed that CNPY2 was significantly increased in RTECs of db/db mice overexpressing CNPY2 (FigureS2A). Compared with db/db control mice, CNPY2‐overexpressing mice exhibited more severe tubular damage, higher serum creatinine and elevated urinary albumin‐to‐creatinine ratio (UACR) levels (Figure S2B,E,F Supporting Information). There were no differences in body weight or blood glucose levels between CNPY2 OE and db/db control mice (Figure S2C,D Supporting Information). Western blot revealed that the expression of CNPY2 and KIM‐1 was notably increased in the CNPY2‐OE group compared to that in the db/db control group (**Figure** **4**A,B). TEM and PLA results revealed that MAM integrity was more severely disrupted by the overexpression of CNPY2 (Figure 4C,D). Moreover, we explored whether the overexpression of CNPY2 affects the level of ferroptosis. As expected, the GSH content was decreased and the levels of MDA and ferrous iron were significantly increased in the kidney tissues of db/db+CNPY2‐OE mice compared with those of db/db+Vehicle and db/db mice (Figure 4E–G). In addition, the cristae of the mitochondria observed by TEM had almost disappeared in the CNPY2‐overexpressing group (Figure 4H). IHC and western blot revealed that TFR‐1 was further upregulated and GPX4 was downregulated in the CNPY‐OE group compared to the db/db control group (Figure 4H–J). The expression levels of ERS proteins, including p‐PERK, ATF4, and CHAC1, were notably higher in the CNPY2‐OE group than in the db/db control group (Figure 4I–J). These results suggest that CNPY2 aggravates renal tubule injury by regulating MAM and ferroptosis.

### 2.5 Knockdown of CPYPY2 Alleviated Renal Tubule Damage in db/db Mice by Inhibiting Ferroptosis and Improving MAM Formation

**Figure 5:**
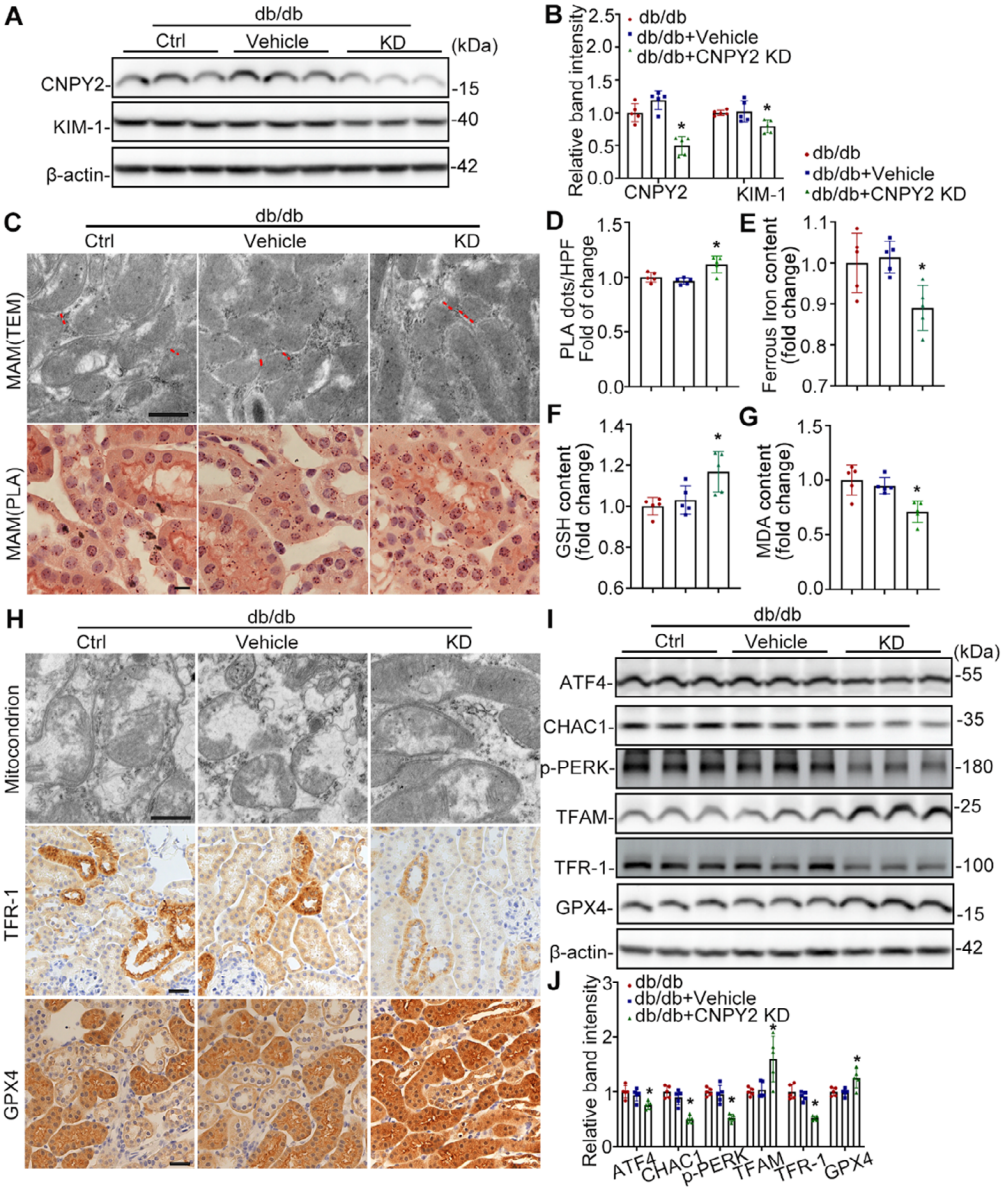
Knockdown of CNPY2 alleviates renal tubule damage in db/db mice by inhibiting ferroptosis and improving MAM formation. A) Western blot bands of CNPY2 and KIM‐1 in the kidneys of db/db mice. B) Quantitative analysis of CNPY2 and KIM‐1 levels in (B), n = 5. C) Representative images of TEM and PLA showing the MAM area. Scale bars, 1 µm (upper panel) and 10 µm (lower panel). D) Quantification of MAM in the PLA images shown in (C), n = 5. E–G) The contents of ferrous iron, GSH, and MDA in the kidneys of db/db mice (n = 5). H) TEM images showing the mitochondrial morphology and IHC staining showing the expression of TFR‐1 and GPX4 in the kidneys of db/db mice. I,J) Western blot bands (I) and quantitative analysis (J) of ATF4, CHAC1, p‐PERK, TFAM, TFR‐1, and GPX4 in the kidneys of db/db mice, n = 5. All the data are expressed as the mean ± SD. ^*^ *P* < 0.05. *P* value was calculated via one‐way ANOVA followed by Tukey's test.

To verify the results of CNPY2 overexpression, we knocked down CNPY2 in RTECs of db/db mice. Immunofluorescence staining showed that CNPY2 was significantly reduced in RTECs of CNPY2‐knocking db/db mice (Figure S3A). Compared with db/db+Vehicle and db/db mice, CNPY2 downregulation led to reduced renal tubule damage, decreased serum creatinine, and significantly lower UACR levels (Figure S3B,E,F Supporting Information). However, CNPY2 knockdown had no significant effect on the body weight or blood glucose levels (Figure S3C,D Supporting Information). The expression of CNPY2 and KIM‐1, as determined by western blot, was strikingly decreased in the kidneys of the CNPY2‐KD group compared to those of the db/db control group (**Figure** **5**A,B). TEM and PLA analyses revealed a substantial increase in MAM area in the kidneys of CNPY2‐deficient mice compared to that in db/db control mice (Figure 5C,D). Additionally, the GSH content was increased and the levels of MDA and ferrous iron were decreased in the kidneys of db/db+CNPY2‐KD mice compared with those of db/db+Vehicle and db/db mice (Figure 5E–G). Moreover, the morphology of the mitochondria was significantly improved, as shown by TEM, in the CNPY2 downregulation group (Figure 5H). IHC and western blot revealed that the expression of TFR‐1 was reduced, and that of GPX4 was elevated in the kidneys of the CNPY‐KD group compared to those of the db/db control group (Figure 5H–J). Compared with the db/db group, the expression levels of ERS pathway‐associated proteins, including p‐PERK, ATF4, and CHAC1, were markedly decreased in the kidneys of the CNPY2 downregulation group (Figure 5I,J).

### 2.6 HG Destroys MAM Integrity and Aggravates Ferroptosis In Vitro

**Figure 6:**
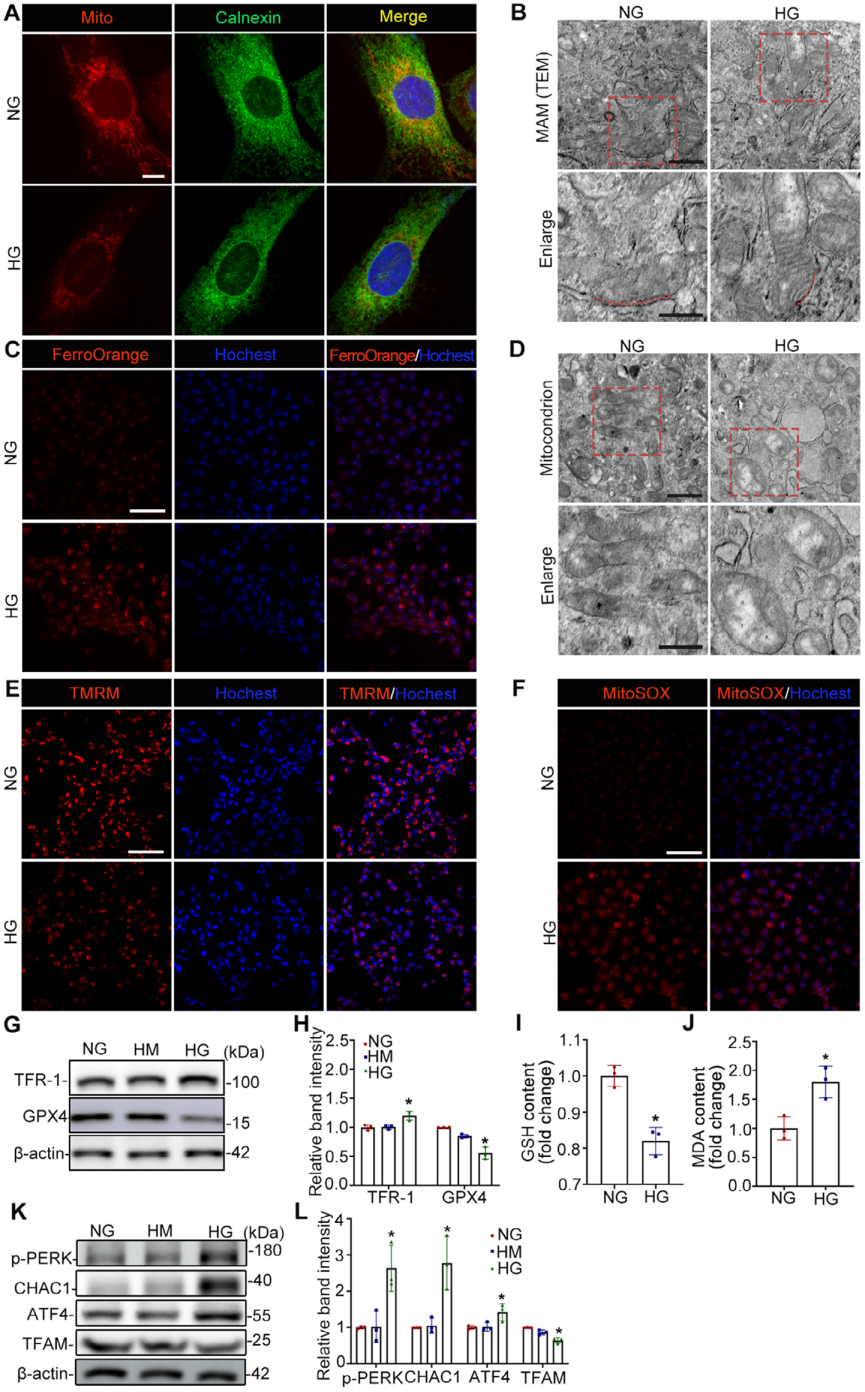
HG destroys MAM integrity and aggravates ferroptosis in vitro. A) Immunofluorescence images of mitochondria (mitoTracker, red) and the ER (calnexin, green) in HK‐2 cells. The nuclei were stained with DAPI. Scale bar = 10 µm. B,D) TEM images showing the MAM area (B) and mitochondrial morphology (D). Scale bars, 1 µm (upper panel) and 500 nm (lower panel). C,E,F) Fe^2+^, mitochondrial membrane potential and mitochondrial ROS in HK‐2 cells were determined using FerroOrange, TMRM, and MitoSOX staining, respectively. Scale bar = 100 µm. G,K) Western blot bands of TFR‐1, GPX4, p‐PERK, CHAC1, ATF4, and TFAM in HK‐2 cells. H,L) Quantitative analysis of western blot bands for (G and K) in HK‐2 cells stimulated with 5.6 mm glucose (NG), mannitol (HM), or 30 mm glucose (HG), n = 3. I,J) The contents of GSH and MDA in HK‐2 cells, n = 3. All the data are expressed as the mean ± SD. *P < 0.05. *P* value was calculated using two‐tailed unpaired *t*‐test (I, J), or one‐way ANOVA followed by Tukey's test (H, L).

Consistent with the findings in vivo, we examined changes in MAM and ferroptosis in HK‐2 cells cultured in HG. As shown in **Figure** **6**A, MAM integrity, co‐staining with mitochondrial (MitoTracker) and ER (Calnexin) markers, was reduced in HG‐stimulated HK‐2 cells. TEM also revealed a significant reduction in the contact surface area between the mitochondria and ER in HG‐treated HK‐2 cells compared with that in the NG group (Figure 6B). Compared with NG, the level of ferrous iron detected by FerroOrange in HG‐activated HK‐2 cells was significantly increased (Figure 6C). The mitochondrial morphology results revealed that the mitochondrial cristae almost disappeared in HG‐stimulated HK‐2 cells (Figure 6D). Consequently, we detected the mitochondrial membrane potential via TMRM and mitochondrial ROS via MitoSOX. As depicted in Figure 6E,F, the mitochondrial membrane potential decreased and mitochondrial ROS were enriched in the HG group. The expression of TFR‐1 was significantly increased, whereas GPX4 expression was notably decreased in HG‐stimulated HK‐2 cells (Figure 6G,H). Compared to the NG group, the GSH content was strikingly decreased, whereas the MDA content was significantly increased (Figure 6I,J). The expression levels of ERS proteins, including p‐PERK, ATF4, and CHAC1, were substantially elevated in HK‐2 cells in the HG environment (Figure 6K,L).

### 2.7 CNPY2 Knockdown Improves MAM Formation and Inhibits Ferroptosis by Targeting PERK in HG‐Stimulated HK‐2 Cells

**Figure 7:**
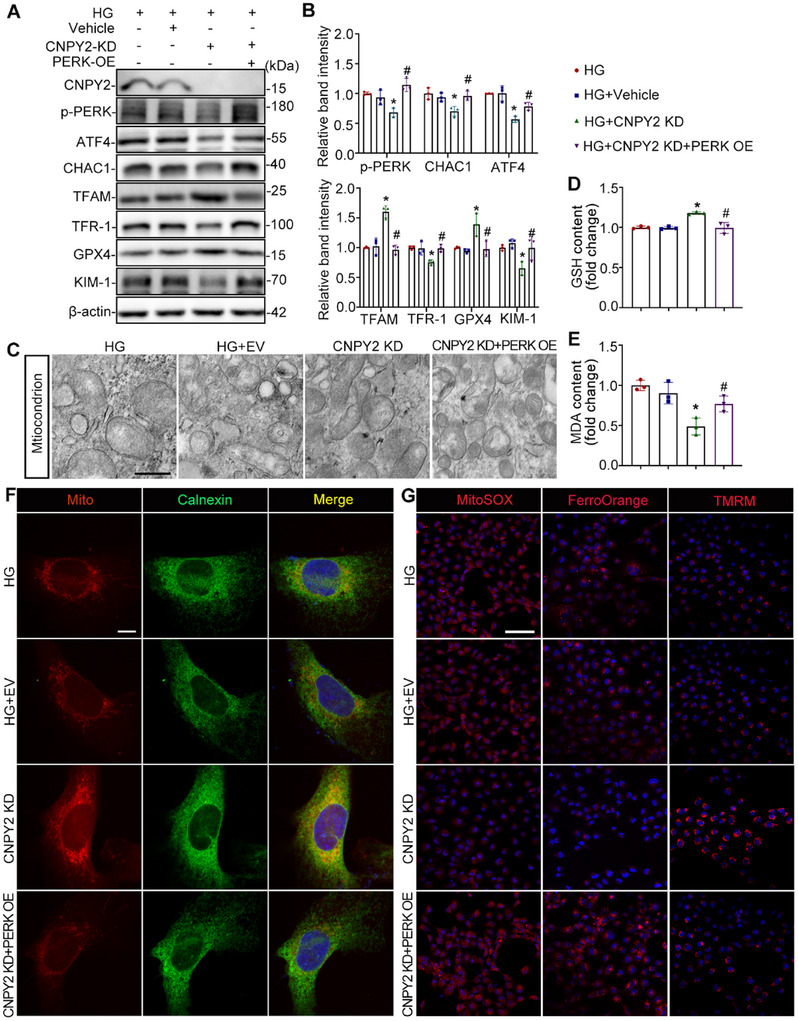
CNPY2 knockdown improves MAM formation and inhibits ferroptosis by targeting PERK in HG‐stimulated HK‐2 cells. A,B) Western blot bands of (A) and quantification analysis of CNPY2, p‐PERK, ATF4, CHAC1, TFAM, TFR‐1, GPX4, and KIM‐1 in HK‐2 cells, n = 3. C) TEM images of mitochondrial morphology in HK‐2 cells. Scale bar = 500 nm. D,E) The contents of GSH and MDA in HK‐2 cells subjected to different treatments, n = 3. F) Immunofluorescence images of mitochondria (mitoTracker, red) and the ER (calnexin, green) in HK‐2 cells. Scale bar = 10 µm. G) Mitochondrial ROS, Fe^2+^ and the mitochondrial membrane potential in HK‐2 cells were determined using MitoSOX, FerroOrange, and TMRM staining, respectively. Scale bar = 100 µm. All the data are expressed as the mean ± SD. Compared to HG and HG+ Vehicle group, *P < 0.05; Compared to HG+CNPY2 KD, ^#^ *P* < 0.05. *P* value was calculated using one‐way ANOVA followed by Tukey's test.

Previous studies have demonstrated that CNPY2 is involved in PERK‐mediated ERS process.^[^ ^17^ ^]^ We next investigated whether CNPY2 knockdown could rescue HG‐induced effects in HK‐2 cells. Compared with the HG and HG+Vehicle groups, ERS‐related and ferroptosis‐related proteins (p‐PERK, ATF4, CHAC1, and TFR‐1) were downregulated, whereas TFAM and GPX4 were upregulated in HK‐2 cells transfected with the CNPY2‐knockdown lentivirus (CNPY2 KD). However, co‐overexpression of PERK reversed the effects of CNPY2 knockdown (**Figure** **7**A,B). TEM revealed that CNPY2 downregulation improved mitochondrial morphology, whereas PERK co‐overexpression aggravated the reduction in mitochondrial cristae (Figure 7C). Compared to the HG and Vehicle groups, downregulation of CNPY2 increased the GSH content and decreased the MDA content in HK‐2 cells in the HG environment. However, co‐overexpression of PERK reversed this effect (Figure 7D,E). Immunofluorescence revealed that the colocalization of mitochondria (MitoTracker) and ER (Calnexin) increased significantly in the CNPY2‐KD group, whereas PERK upregulation in HK‐2 cells with CNPY2‐KD decreased this colocalization (Figure 7F). Finally, MitoSOX, FerroOrange, and TMRM staining revealed that the knockdown of CNPY2 in an HG environment decreased the levels of mitochondrial ROS and ferrous iron and increased the mitochondrial membrane potential, which was counteracted by the co‐overexpression of PERK (Figure 7G).

### 2.8 CNPY2 Overexpression Exacerbates MAM Integrity and Ferroptosis by Targeting PERK in HK‐2 Cells Under HG Conditions

**Figure 8:**
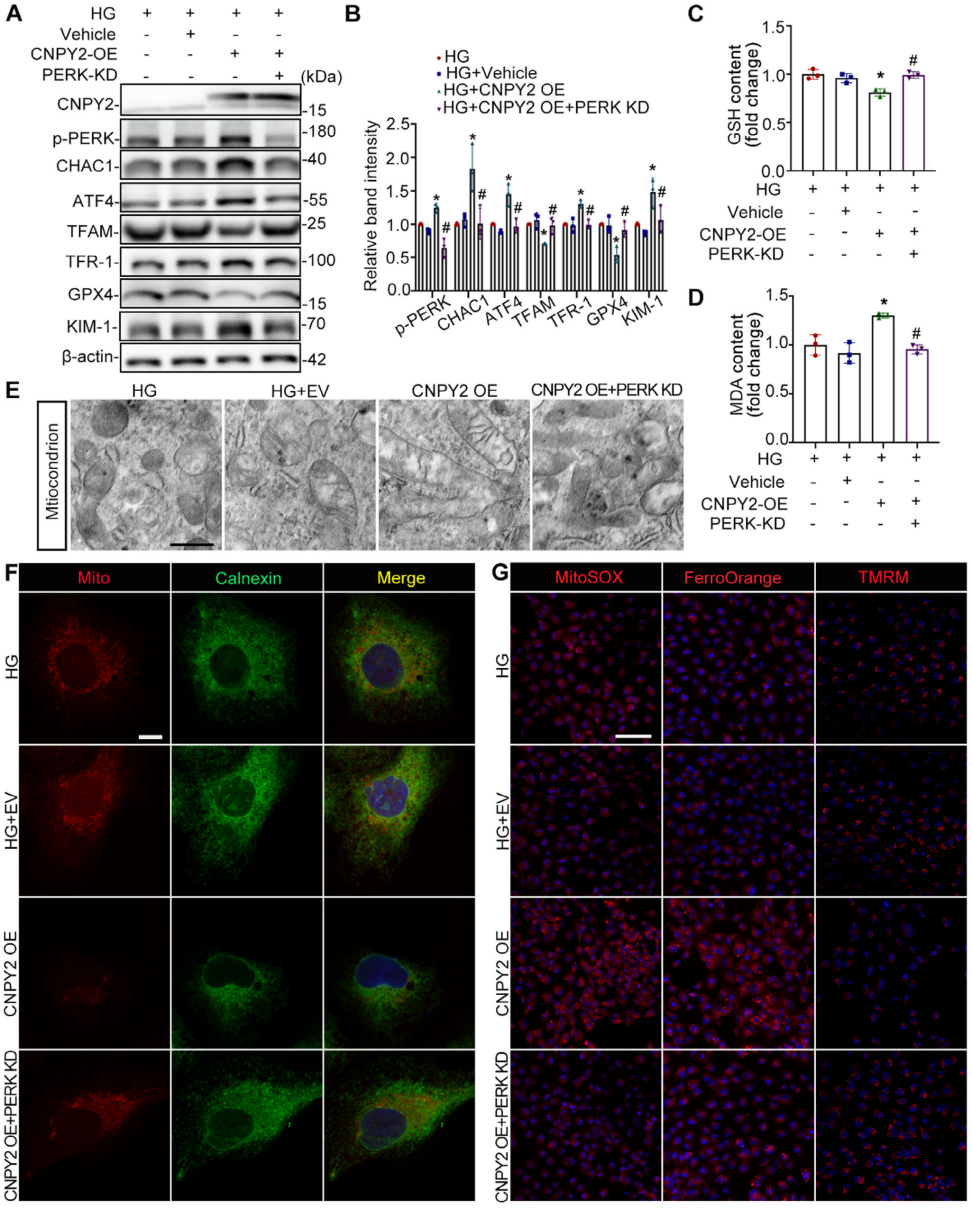
CNPY2 overexpression exacerbates MAM integrity and ferroptosis by targeting PERK in HK‐2 cells under HG conditions. A,B) Western blot bands of (A) and quantification analysis of CNPY2, p‐PERK, ATF4, CHAC1, TFAM, TFR‐1, GPX4, and KIM‐1 in HK‐2 cells, n = 3. C,D) The contents of GSH and MDA in HK‐2 cells subjected to different treatments, n = 3. E) TEM images of mitochondrial morphology in HK‐2 cells. Scale bar = 500 nm. F) Immunofluorescence images of mitochondria (mitoTracker, red) and the ER (calnexin, green) in HK‐2 cells. Scale bar = 10 µm. G) MitoSOX, FerroOrange, and TMRM staining were used to determine the levels of mitochondrial ROS, Fe^2+^ and the mitochondrial membrane potential in HK‐2 cells. Scale bar = 100 µm. All the data are expressed as the mean ± SD. Compared to HG and HG+ Vehicle group, ^*^ *P* < 0.05; compared to HG+CNPY2 OE group, ^#^ *P* < 0.05. *P* value was calculated using one‐way ANOVA followed by Tukey's test.

We overexpressed CNPY2 in HG‐induced HK‐2 cells to clarify whether it aggravated HK‐2 cell injury via PERK. As shown in **Figure** **8**A,B, the expression of p‐PERK, ATF4, CHAC1, and TFR‐1 increased, whereas the expression of TFAM and GPX4 decreased significantly in HK‐2 cells transfected with CNPY2‐overexpressing lentivirus (CNPY2 OE); however, downregulation of PERK rescued the expression of these proteins. Moreover, the GSH content decreased and the MDA content increased in the CNPY2‐OE group compared to those in the HG and vehicle groups. Similarly, the co‐downregulation of PERK increased the GSH content and reduced the MDA content (Figure 8C,D). TEM also revealed that CNPY2 overexpression resulted in fewer mitochondrial cristae, whereas PERK co‐knockdown protected mitochondrial morphology (Figure 8E). There was less colocalization of mitochondria (MitoTracker) and ER (Calnexin) in the CNPY2‐OE group than in the HG and Vehicle groups, whereas PERK knockdown in HK‐2 cells with CNPY2‐OE led to the opposite trend (Figure 8F). MitoSOX, FerroOrange, and TMRM staining showed that mitochondrial ROS and Fe^2+^ levels were strongly elevated and mitochondrial membrane potential levels were dramatically decreased in the context of CNPY2 overexpression; however, these damaging effects were reversed by PERK knockdown (Figure 8G).

## 3 Discussion

Ferroptosis is characterized by iron overload and accumulation of lipid peroxidation,^[^ ^20^ ^]^ contributing to the pathological processes of various metabolic disorders,^[^ ^8^, ^21^, ^22^ ^]^ including DN.^[^ ^23^, ^24^ ^]^ Kim et al.^[^ ^10^ ^]^ assessed ferroptosis in streptozotocin‐induced diabetic mice and RTECs cultured with HG in vitro. This study identified the characteristic pathological changes associated with ferroptosis, such as reduced GSH content, elevated MDA and Fe^2+^ levels, and loss of mitochondrial cristae. In this study, we examined the alterations in ferroptosis within the renal tubules of patients with DN, db/db mice, and HK‐2 cells exposed to HG conditions. Consistent with previous findings, we observed significant upregulation of TFR‐1 expression, along with a marked reduction in both GPX4 expression and GSH content. Additionally, there was a notable increase in MDA and ferrous iron levels in the RTECs of DN. Furthermore, changes in mitochondrial morphology, characterized by the reduction or loss of mitochondrial cristae, were evident. These findings underscore the critical role of ferroptosis in DN pathophysiology.

MAM serves as a crucial hub for ER and mitochondrial connectivity, facilitating the exchange of lipids, Ca^2+^, and other signaling molecules.^[^ ^25^ ^]^ It is important to maintain a distance of no less than 10 nm and no more than 30 nm between the ER and the outer mitochondrial membrane,^[^ ^26^ ^]^ which is necessary for effective protein interactions and substance exchange. Numerous studies have indicated that MAM dysfunction plays a pivotal role in the progression of DN.^[^ ^27^, ^28^ ^]^ Xie et al. reported that RTN1A, which resides in the ER, is overexpressed in DN renal tubules and aggravates ERS and mitochondrial dysfunction in DN by regulating MAM.^[^ ^14^ ^]^ Similarly, another study demonstrated that the destruction of MAM formation, ERS, mitochondrial dysfunction, and tubular epithelial cell injury occurred in RTECs under HG conditions. Overexpression of PCAS‐2, a MAM interface anchor protein, mitigated renal tubule injury in diabetic mice by improving the integrity and stability of MAM.^[^ ^29^ ^]^ Our study also verified that MAM formation, detected by PLA and TEM, was disrupted in the RTECs of patients with DN, as well as in db/db mice. Notably, we found that CNPY2 may disrupt MAM integrity by promoting PERK phosphorylation.

CNPY2, a newly discovered transmembrane protein located in the ER, is a key promoter of the unfolded protein reaction, and is mainly involved in ERS.^[^ ^17^ ^]^ In hepatocarcinoma, CNPY2 is highly expressed and participates in the occurrence and development of hepatocarcinoma by inhibiting and activating oncogenes via unfolded protein reaction‐dependent p53.^[^ ^30^ ^]^ Zhao et al.^[^ ^31^ ^]^ reported that in a rat model of ischemic stroke, berberine improved neuronal apoptosis by inhibiting CNPY2‐induced ERS overactivation. In rat models of myocardial ischemia‐reperfusion injury, activation of CNPY2 downstream of the PERK pathway led to cardiomyocyte apoptosis and injury.^[^ ^32^ ^]^ Another study^[^ ^33^ ^]^ reported that the renin‐angiotensin system activated CNPY2/PERK‐mediated mitochondrial division and promoted endothelial cell apoptosis in hypertensive mouse models. These studies suggest that targeting CNPY2 in various diseases is a potential therapeutic approach. However, whether and how CNPY2 regulates renal tubular injury in DN has not yet been reported. The most compelling finding of our study was the potential contribution of CNPY2 upregulation to kidney tubular injury in patients with DN and db/db mice. Previous studies have demonstrated that HG‐induced mitochondrial dysfunction, reduction in MAM, and increased apoptosis in vitro are associated with downregulation of mitofusin 2 (Mfn2) and activation of the PERK pathway.^[^ ^34^ ^]^ In the current study, we observed that CNPY2 activates the PERK pathway, leading to MAM disruption and ferroptosis in vitro. Furthermore, inhibition of PERK expression by lentivirus transfection in HG‐stimulated HK‐2 cells improved MAM formation and alleviated ferroptosis.

Previous studies suggested that PERK/ATF4‐related pathways are involved in ferroptosis.^[^ ^35^, ^36^, ^37^ ^]^ As a component of the unfolded protein reaction, CHAC1 is the downstream regulatory molecule of ATF4, which induces ferroptosis by regulating GSH depletion and promoting the occurrence and development of various diseases.^[^ ^38^, ^39^, ^40^ ^]^ A recent study indicated that short‐term methionine starvation promoted ferroptosis in cancer cells by stimulating CHAC1 transcription and depleting intracellular GSH, thereby increasing the efficacy of cancer immunotherapy.^[^ ^41^ ^]^ Bioinformatics analysis revealed that CHAC1 is a key gene in the process of ferroptosis in ischemic stroke, and further in vivo experiments revealed that the application of anti‐CHAC1 exosomes eased cerebral ischemia‐reperfusion injury by inhibiting ferroptosis.^[^ ^42^ ^]^ Our findings also indicated that the overexpression of CNPY2 in a high‐glucose environment resulted in the phosphorylation of PERK, subsequently activating the downstream expression of ATF4 and enhancing the transcription of CHAC1. This sequence of events led to the depletion of intracellular GSH, reduction in GPX4 expression, and an imbalance in redox homeostasis. Unfortunately, we did not conduct interventions targeting CHAC1 to verify the alterations in ferroptosis in this study.

Taken together, our findings demonstrate, for the first time, that ER‐related CNPY2 facilitates kidney tubule injury in DN by modulating ferroptosis and MAM. This suggests that CNPY2 could serve as a promising therapeutic target for patients with DN; however, its precise role in DN requires further investigation. Consequently, we intend to further investigate the underlying mechanisms involving CNPY2, MAM, and ferroptosis in DN pathogenesis to aid in the development of effective therapeutic strategies in the future.

## 4 Experimental Section

### Patients and Samples

Human renal biopsy samples were obtained from the First Affiliated Hospital of Zhengzhou University. This study was approved by the Institutional Ethics Committee of the First Affiliated Hospital of Zhengzhou University. All patients provided informed consent (Ethics Approval No. 2023‐KY‐0501‐004). All participant information is presented in Table S1 (Supporting Information).

### Animal Studies

All mouse experiments were approved by the Animal Care and Use Committee of Zhengzhou University (ZZU‐LAC20230526^[^ ^26^ ^]^). Six‐week‐old male db/db diabetic mice (n = 40) and db/m control mice (n = 6) were purchased from GemPharmatech (Nanjing, China) and housed at the Laboratory Animal Center of Zhengzhou University. All the animals were maintained under standard husbandry conditions.

Male db/db mice aged 10 weeks were randomly divided into the following groups: 1) db/db group (n = 8), in which the mice were fed a normal diet every day without special treatment; 2) db/db + CNPY2‐KD group (n = 8), in which the mice received ksp‐cadherin promoter adeno‐associated virus (AAV)‐9 carrying the specific mouse CNPY2 shRNA (Table S2, Supporting Information) treatment via tail‐vein injection at week 10 and 16, respectively; 3) db/db + Vehicle group (CNPY2‐KD control, n = 8), in which the mice received adeno‐associated virus vector treatment via tail‐vein injection; 4) db/db + CNPY2‐OE group (n = 8), in which the mice received ksp‐cadherin promoter adeno‐associated virus (AAV) carrying the full‐length mouse CNPY2 (AAV9‐CNPY2) via tail‐vein injection at week 10 and 16, respectively; 5) db/db + Vehicle group (CNPY2‐OE control, n = 8), in which the mice received adeno‐associated virus vector treatment via tail‐vein injection. All AAVs were purchased from GeneChem (Shanghai, China). All mice were euthanized at 22 weeks of age and their kidneys were rapidly collected.

### Cell Culture and Transfection

Human proximal tubular epithelial cells (HK‐2) were obtained from QuiCell Biotechnology (H351; QuiCell, China) and cultured in DMEM + F12 medium (#11966‐025, #11765‐054, Gibco, USA) supplemented with 10% fetal bovine serum (#10099141; Gibco, USA) and 1% penicillin‐streptomycin (#PB180120; Procell, China). The cells were treated with normal glucose (NG, 5.6 mm glucose) or high glucose (HG, 30 mm glucose) and transfected with lentiviruses (Genechem, Shanghai, China) to overexpress CNPY2 or knockdown CNPY2 and PERK (Table S2, Supporting Information). The PERK overexpression plasmids obtained from GeneChem (Shanghai, China) were transfected with Lipofectamine 3000 (L3000008, Thermo Fisher Scientific, USA).

### Western Blot

Renal tissues and HK‐2 cells were lysed in a suitable volume of RIPA buffer (#R0010, Servicebio, China) containing phosphatase inhibitors (#cw2383S, CWBio, Beijing, China) and PMSF (#p0100, Servicebio, Beijing, China). Proteins were subjected to electrophoresis and transferred to PVDF membranes. After blocking with 5% skim milk, the membranes were incubated with primary antibodies (listed in Table S3, Supporting Information) at 4 °C overnight. The following day, membranes were incubated with secondary antibodies (#A21020 or # A21010; Abbkine, Wuhan, China) for 1 h. Proteins in the membranes were detected using enhanced chemiluminescence (ECL) buffer on an AI600 imager (Thermo Fisher Scientific, USA).

### Transmission Electron Microscopy (TEM)

Kidney tissues and HK‐2 cells were fixed with 2.5% glutaraldehyde and post‐fixed with 1% osmium tetroxide. After dehydration and embedding, the samples were coated with 812 epoxy resin and polymerized at 60 °C. Ultrathin sections were cut into 60 nm sections and stained with uranyl acetate and lead citrate. The images were obtained using a TEM (Hitachi, Tokyo, Japan).

### Hematoxylin–Eosin (HE) Staining, Periodic acid Schiff (PAS) Staining, and Immunohistochemical (IHC) Staining

Mouse kidney tissues were embedded in paraffin and cut into 3 µm sections; subsequently, HE and PAS staining were performed according to the kit protocols (Solarbio, Beijing, China). For IHC staining, kidney tissue sections were subjected to antigen retrieval and blocked with 5% bovine serum albumin. The sections were subsequently incubated with primary antibodies (listed in Table S3, Supporting Information) at 4 °C overnight. The next day, the secondary antibody was added. Images were obtained using a light microscope (Olympus, Tokyo, Japan).

### In Situ PLA

After dewaxing, antigen repair, permeabilization, and blocking, paraffin‐embedded kidney sections were incubated with VDAC1 and IP3R1 antibodies (listed in Table S3, Supporting Information) at 4 °C overnight. After washing, the kidney sections were incubated with anti‐rabbit PLA probe Plus and anti‐mouse PLA probe Minus (#DUO92002 and #DUO92004, Sigma–Aldrich, USA) for 1 h at 37 °C. The sections were subsequently subjected to ligation and amplification using the Duolink assay with bright‐field detection (#DUO92012, Sigma–Aldrich, USA). Finally, images were captured using a light microscope (Olympus, Tokyo, Japan).

### Detection of the Contents of MDA, GSH, and Ferrous rion

The levels of MDA, GSH, and Ferrous iron in kidney tissues and HK‐2 cells were detected using an MDA assay kit (S0131S, Beyotime, Jiangsu, China), GSH assay kit (BC1175, Solaibao, Beijing, China), and Ironassay kit (#MAK025, Sigma–Aldrich, USA), respectively, following the manufacturer's protocol.

### Measurement of Mitochondrial ROS, Ferrous iron, and the Mitochondrial Membrane Potential in HK‐2 Cells

The levels of mitochondrial ROS, Ferrous ironand the mitochondrial membrane potential in HK‐2 cells were assessed by staining via the MtSOX Deep Red assay (MT14, Dojindo, Japan), FerroOrange (F374, Dojindo, Japan) and Image‐iT TMRM (I34361, Invitrogen, USA), following the manufacturer's instructions, and fluorescence images were captured via a confocal microscope (Carl Zeiss, Germany).

### Immunofluorescence Assay

For immunofluorescence, kidney tissue sections were antigen‐repaired, permeabilized with 0.1% Triton X‐100, and blocked with 5% bovine serum albumin. The sections were subsequently incubated with primary antibodies (listed in Table S3, Supporting Information) at 4 °C overnight. For immunofluorescence, HK‐2 cells were seeded onto confocal dishes and stained with MitoTracker Red (M7512; Invitrogen, USA) for 30 min. The cells were then fixed, permeabilized, and blocked. Finally, the cells were incubated at 4 °C with anti‐Calnexin (Table S3, Supporting Information) overnight. The next day, the kidney sections or HK‐2 cells were incubated with Alexa Fluor Plus 488 secondary antibody (1:400, A32731, Thermo Fisher Scientific, USA). Fluorescence images were obtained using a confocal microscope (Carl Zeiss, Germany).

### Statistical Analyses

Statistical comparisons between the two groups were performed using an unpaired *t* test with GraphPad Prism 8.0. For three or more groups, one‐way ANOVA followed by Tukey's test was used. The data are presented as the mean ± standard deviation (SD), with *P* < 0.05 indicating statistical significance.

## Conflict of Interest

The authors declare no conflict of interest.

## Author Contributions

Z.L. and Q.F. supervised and designed the study. J.C. and D.L. wrote the original manuscript. J.C., D.L., L.L.,T.L., S.P., H.W., and Y.Q. performed experiments. J.C., Y.L., and L.L. contributed to data acquisition and analysis. J.C., Z.L., and Q.F revised the manuscript.

## Supporting information



Supporting Information

## Data Availability

The data that support the findings of this study are available from the corresponding author upon reasonable request.
