# Supplementary material for: CNPY2 Aggravates Renal Tubular Cell Ferroptosis in Diabetic Nephropathy by Regulating PERK/ATF4/CHAC1 Pathway and MAM Integrity
Source: Adv Sci (Weinh). 2025 Apr 11;12(25):2416441. doi: 10.1002/advs.202416441 (PMC12224942; doi:10.1002/advs.202416441)
Supplement: Supplementary file 1 — Supporting Information [file ADVS-12-2416441-s001.docx]

**Supplementary Information**

**CNPY2 Aggravates Renal Tubular Cell Ferroptosis in Diabetic Nephropathy by Regulating PERK/ATF4/CHAC1 Pathway and MAM Integrity**

The supplementary file includes 3 Table and 3 Figures.

**Supplementary Table S1. Clinical characteristics of control subjects and patients with DN**

|  | Control (n=11) | DN (n=22) |
| --- | --- | --- |
| Male (%) | 7 (63.6) | 17 (77.2) |
| Age (years) | 46.82±10.30 | 55.36±4.836 |
| HbA1c (%) | 5.47±0.57 | 6.75±1.52 |
| Scr (µmol/L) | 71.2±11.17 | 149.0 (134.5, 256.0) ^*^ |
| BUN (mmol/L) | 4.55±0.69 | 11.78 (8.85, 17.14) ^*^ |
| eGFR (mL/min/1.73m^2^) | 101.0±9.32 | 39.6 (23.30, 48.70) ^*^ |
| UACR (g/g) | 0.02 (0.02) | 2.96±2.15^*^ |

HbA1c, hemoglobin A1c; UA, Uric Acid; Scr, serum creatinine; BUN, blood urea nitrogen; eGFR, estimated glomerular filtration rate; UACR, urine albumin to creatinine ratio. Data are plotted as the mean ± SD. **P* < 0.05.

**Supplementary Table S2. The sequence of shRNA.**

| Genes | Supplier | Sequence |
| --- | --- | --- |
| mouse-*Cnpy2* | Genechem | GAGAGGCTGACAACGTTAAAG |
| human-*CNPY2* | Genechem | GATGGGATCTTTCCGGATCAA |
| human-*PERK* | Genechem | CCTCAAGCCATCCAACATATT |

**Supplementary Table S3. The detailed information of the antibodies used in this study**

| Antibodies | Supplier | Catalog number | Dilution |
| --- | --- | --- | --- |
| CNPY2 | Proteintech | 14635-1-AP | WB 1:1000  IHC 1:200  IF 1:100 |
| ATF4 | Proteintech | #60035-1-Ig | WB 1:2000 |
| CHAC1 | Proteintech | 15207-1-AP | WB 1:1000 |
| TFAM | Proteintech | 22586-1-AP | WB 1:5000 |
| PERK | Proteintech | 24390-1-AP | WB 1:1000 |
| VDAC1 | Proteintech | 66345-1-Ig | IHC 1:500 |
| IP3R1 | Proteintech | 19962-1-AP | IHC 1:200 |
| Calnexin | Proteintech | 10427-2-AP | IF 1:200 |
| p-PERK | Affinity Biosciences | DF7576 | WB 1:1000 |
| GPX4 | Abcam | ab125066 | WB 1:1000  IHC 1:800 |
| TFR-1 | Abcam | ab214039 | WB 1:1000  IHC 1:400 |
| GRP78 | Abcam | ab21685 | WB 1:1000 |
| 4-HNE | Thermo Fisher Scientific | MA5-27570 | IF 1:25 |
| Lotus Tetragonolobus Lectin (LTL) | Vectorlabs | FL-1321-2 | IF 1:200 |
| KIM-1 | Novus Biologicals | NBP1-76701 | WB 1:1000  IHC 1:200 |
| β-Actin | ZEN-BIOSCIENCE | 200068-8F10 | WB 1:5000 |


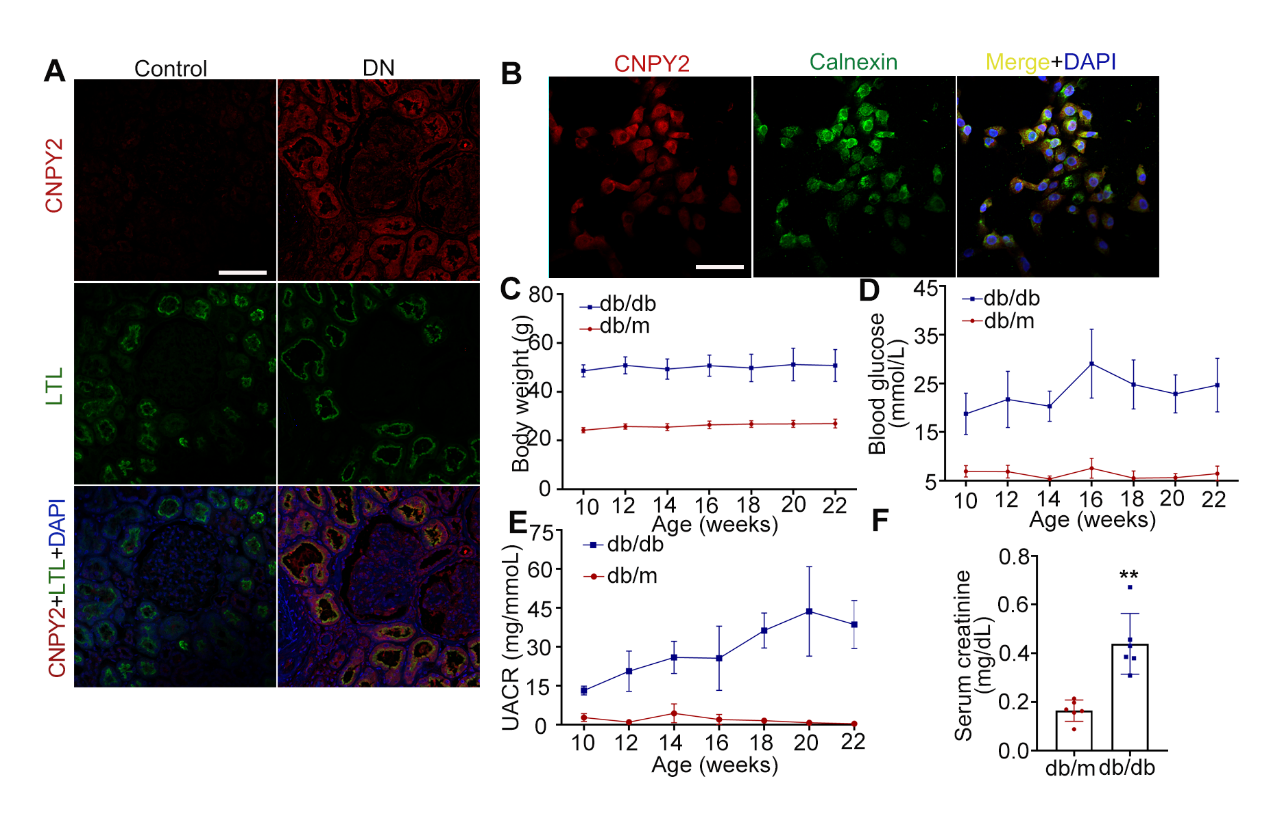


**Supplementary Fig. 1 CNPY2 was mainly localized in kidney tubular cells.** **(A)** Co-immunostaining shows the localization of CNPY2 in the kidneys of patients with DN. The kidneys were double stained with Cnpy2 (red), and LTL (green). The nuclei were stained with DAPI (blue). Scale bar =100 µm. **(B)** Representative images of immunofluorescence staining for colocalisation of CNPY2 (red) with a endoplasmic reticulum biomarker (Calnexin, green) in HK-2 cells. The nucleus was stained with DAPI. Scale bar =100 µm. **(C)** Body weight, **(D)** Blood glucose levels, **(E)** The levels of UACR and **(F)** Scr in the db/m and db/db mice, n=5-6. All data are expressed as the mean ± SD. ***P* < 0.01. *P* value was calculated by two-tailed unpaired t-test.


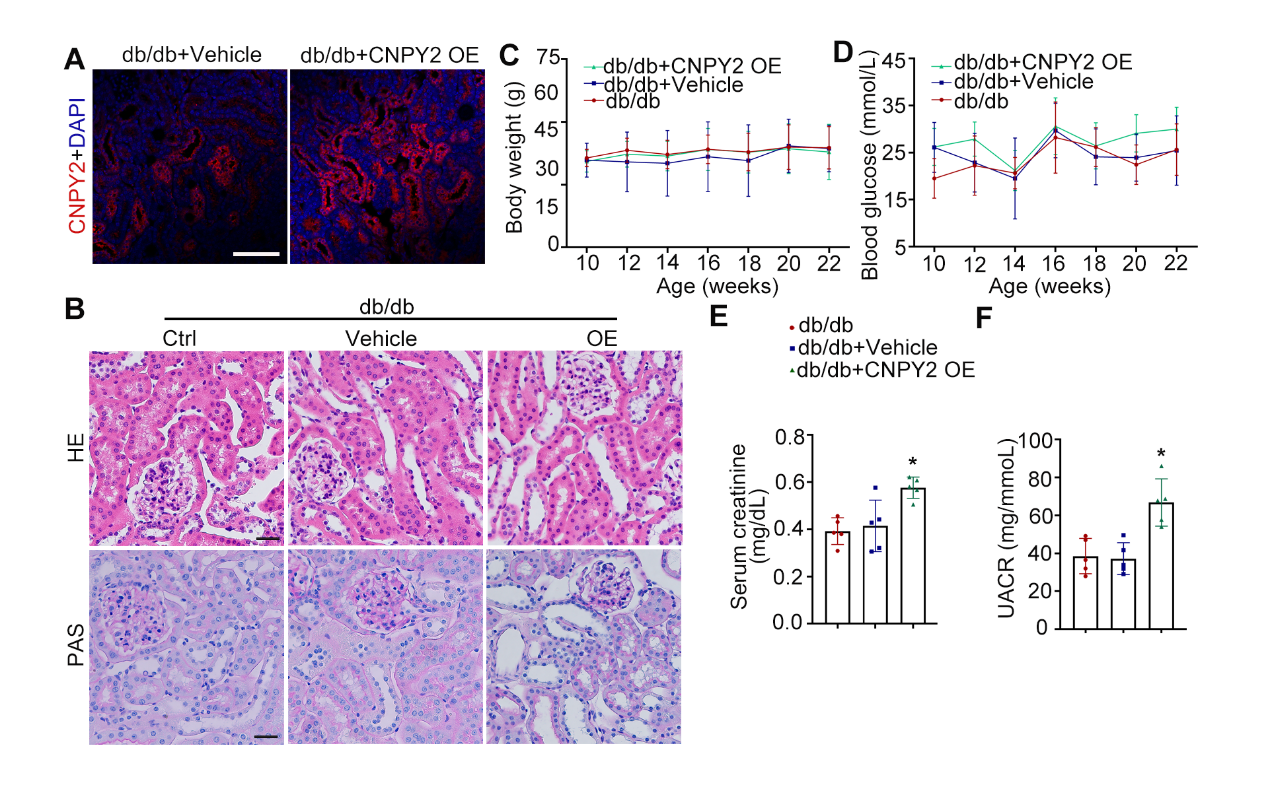


**Supplementary Fig. 2 Overexpression of CNPY2 aggravates the pathological changes of db/db mice.** **(A)** Representative images of immunofluorescence staining in the kidney tissues of db/db mice. Scale bar =100 µm. **(B)** HE and PAS staining of kidney tissues in db/db mice. Scale bar =50 µm. (**C)** Body weight, (**D)** Blood glucose levels **(E)** The levels of Scr and **(F)** UACR in the different groups of mice, n=5. All the data are expressed as the mean ± SD. **P* < 0.05. *P* value was calculated using one-way ANOVA followed by Tukey’s test (E-F).


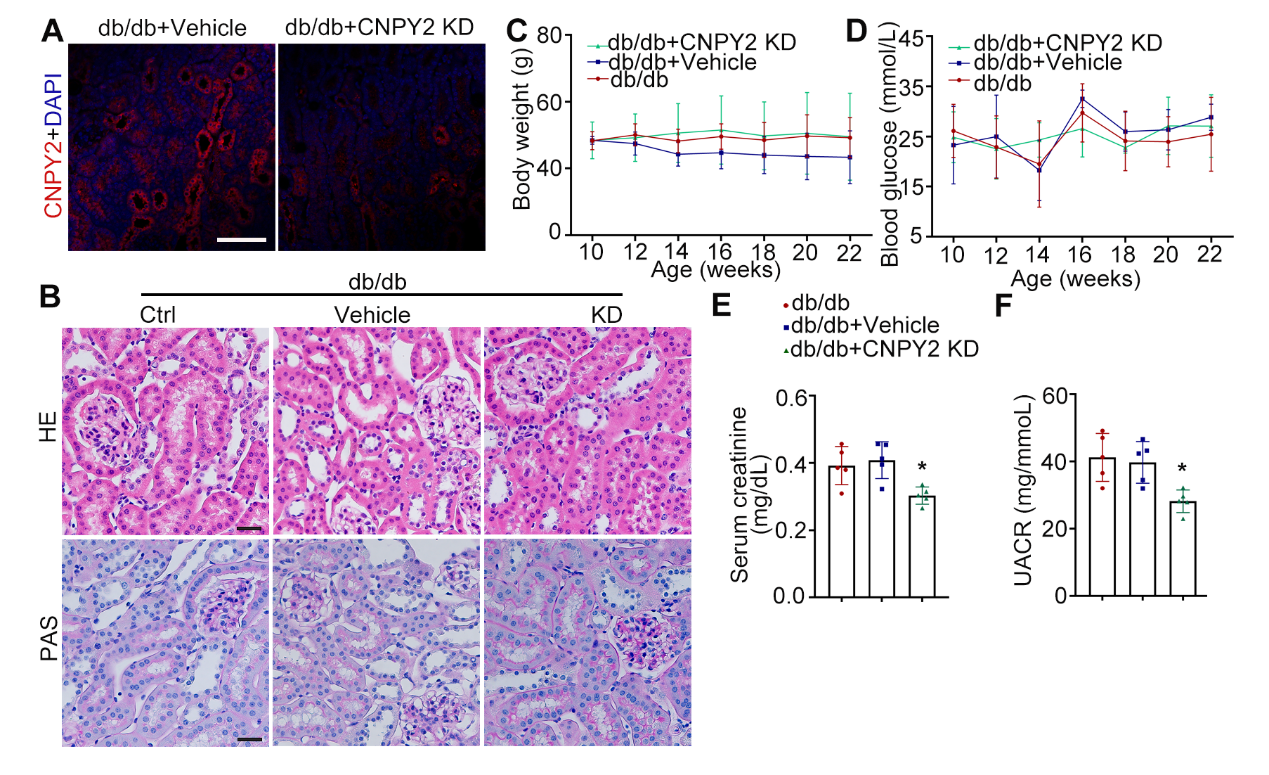


**Supplementary Fig. 3 Knockdown of CNPY2 alleviates the pathological changes of db/db mice.** (A) Representative images of immunofluorescence staining in the kidney tissues of db/db mice. Scale bar =100 µm. (B) HE and PAS staining of kidney tissues in db/db mice. Scale bar =50 µm. (C) Body weight, (D) Blood glucose levels (E) The levels of Scr and (F) UACR in the different groups of mice, n=5. All the data are expressed as the mean ± SD. *P < 0.05. P value was calculated using one-way ANOVA followed by Tukey’s test (E-F).
